# A realist synthesis of websites containing content on perfectionism: Are the descriptions and advice empirically supported?

**DOI:** 10.1186/s40359-021-00620-8

**Published:** 2021-08-25

**Authors:** Tracey D. Wade, Sarah J. Egan, Maggie Wleklinski, Amy O’Brien, Grace Fitzallen, Roz Shafran

**Affiliations:** 1 Órama Institute and Blackbird Initiative, Flinders University, Adelaide, SA Australia; 2 Discipline of Psychology, School of Population Health, Curtin University, Perth, Australia; 3 University College London, Great Ormond Street Institute of Child Health, London, England

**Keywords:** Perfectionism, Youth, Depression, Anxiety, Websites

## Abstract

**Background:**

Perfectionism is a risk factor for depression and anxiety and is increasing in young people. It is important to understand the information that youth are exposed to about perfectionism on the internet and what may be required to make this more helpful in terms of accessing empirically supported descriptions and advice.

**Methods:**

This research used novel methodology to investigate content about perfectionism on websites by conducting a realist synthesis of the definitions of perfectionism, and the degree to which websites contain empirically supported strategies and recognise the advantages and disadvantages of perfectionism. The results were presented to people aged 18 to 24 (N = 18) with a lived experience of anxiety/depression for feedback.

**Results:**

The search yielded 992 websites, 266 of which were included in the synthesis; only one met the criteria for excellent quality with most (56%) judged as moderate. The feelings, thoughts, and behaviours that accompany perfectionism were commonly described, and strategies included identifying cognitions and developing alternatives, moving from self-criticism to self-compassion, normalising mistakes, adjusting goals, receiving practical support, and strategies for procrastination. The young people wanted further emphasis on depression and anxiety as consequences of perfectionism that contributed to a vicious cycle. They identified interventions were difficult, with greater levels of support needed.

**Conclusions:**

While most websites contained empirically supported information, the quality needs to improve, and further information needs to be provided on the links with anxiety and depression. Interventions for perfectionism need to have more focus on helping young people develop support networks.

## Background

### Definition of perfectionism

The most frequently used measures of perfectionism focus on high standards and striving to be the best, and concern over mistakes and gaps between desired and actual performance, and perceptions of other’s expectations about performance [1, 2]. The definition and model of clinical perfectionism, derived to guide cognitive behaviour therapy for perfectionism (CBT-P), recognises the overdependence of self-worth based on striving to meet personally demanding standards despite negative consequences [3], where self-criticism when standards are not met reinforces self-evaluation dependent on striving and achievement. The most common definitions and associated measures of perfectionism are shown in Table 1. Two main dimensions are typically recognised, an unrelenting pursuit of high and rigid standards, and overidentification of self-worth and identity with the attainment of these standards. Perfectionism has linearly increased in youth over 1989–2017 [4].

**Table 1:** Definitions and measures of perfectionism and self-criticism

Concept	Definition	Common measures
Clinical perfectionism	Core psychopathology is the overdependence of self-evaluation on the determined pursuit of personally demanding, self-imposed standards in at least one highly salient domain despite adverse consequences	Clinical Perfectionism Questionnaire [3] a 12- item scale. *Example items*: Have you been told that your standards are too high? Have you felt a failure as a person because you have not succeeded in meeting your goals?
Perfectionism	Perfectionism is typically viewed as having two dimensions, striving towards perfectionistic standards and perfectionistic concerns. A definition which captures both dimensions is: “Those whose standards are high beyond reach or reason, people who strain compulsively and unremittingly toward impossible goals and who measure their own worth entirely in terms of productivity and accomplishment” (Burns, 1980)	Frost Multidimensional Perfectionism Scale [1] the two most widely used subscales are Personal Standards (7 items) and Concern over Mistakes (9 items). *Respective example items*: I hate being less than the best at things; The fewer mistakes I make, the more people will like me Hewitt and Flett Multidimensional Perfectionism Scale [2] the two most widely used subscales are Self-oriented Perfectionism and Socially Prescribed Perfectionism. Respective example items: have you been told that your standards are too high? My family expects me to be perfect

### Effective interventions for perfectionism

Perfectionism is strongly related to anxiety, depression and disordered eating across the developmental spectrum [5–7] and has become a treatment target across different psychopathologies [8]. Meta-analytic evidence shows that CBT-P produces large and medium effect size decreases in perfectionism/disordered eating and depression/anxiety respectively [9]. Between group effect sizes range from 0.48 to 0.55 for perfectionism, 0.62 for depression, 0.49 for anxiety [10], and 0.64 for disordered eating [11]. The most common elements of CBT-P [12], informed by the definition and model of clinical perfectionism, are shown in Table 2 i.e., psychoeducation related to achievement, how perfectionistic and rigid goals and self-criticism can interfere with approaching valued goals, broadening the factors that impact on self-worth to include those unrelated to achievement.

**Table 2:** Content for each session of internet delivered CBT-P

Session #	Topic
1	Defining perfectionism and identifying maintaining factors
2	Individualized formulation of perfectionism
3	Enhancing motivation to change perfectionism
4	Psychoeducation, self-monitoring and surveys
5	Behavioural experiments, challenging dichotomous thinking
6	Challenging unhelpful thinking styles
7	Procrastination, time management, pleasant events
8	Self-criticism versus self-compassion, self-evaluation and relapse prevention

These interventions are scalable. They have been delivered in classroom settings (mean age of 14 years), producing significantly lower perfectionism, self-criticism and depression/anxiety than controls at 6-month follow-up [13]. They are as effective when delivered as an internet intervention compared to face-to-face [10], and result in significant and moderate reductions in depression and anxiety in young adults with a mean age of 26 years [14] and youth aged 14–19 years [15].

### Aims

The research-practice gap (dissemination of evidence-based interventions from controlled research environments to routine clinical care or community settings) is widely recognised as problematic [16]. Despite clear evidence for effective internet interventions for perfectionism in young people [13–15], there has long been concern about using the web for *information* about mental health, in terms of too much information and no quality control [17]. We hypothesised that internet sites providing information about perfectionism may not be clearly providing accurate advice on evidence-based strategies and interventions. Our first aim was to therefore investigate the content of internet sites that present information on perfectionism, using a realist synthesis, to identify: (1) definitions of perfectionism and how various elements of perfectionism might work together to increase anxiety and depression, (2) the degree to which the websites contain evidence-based strategies for targeting perfectionism in a way that may decrease anxiety and depression in young people, and (3) whether websites recognised one of the main barriers to successful adoption of helpful strategies, namely the ambivalence attending perfectionism, with perceived advantages and disadvantages. Realist synthesis research uses an explanatory rather than judgemental focus that seeks to ‘unpack the mechanisms’ which determine when complex interventions work best [18]. Our second aim was to incorporate lived experience of young people who are particularly vulnerable to perfectionism, in order to increase the usefulness of research to meet critical evidence gaps [19]. Specifically, we consulted with youth with lived experience of depression and anxiety about the information on the websites, with a view to both informing ways to make this information more useful, as well as to inform future iterations of development of CBT-P that can better meet the needs of youth.

## Method

### Search strategy

DEVONagent for Mac was used to search across websites using the following customised Search Set: Language: International; Ignore Diacritics checked; Fuzzy checked (allow alternate spelling); Similar pages NOT filtered; Archived pages NOT filtered; Searches terms in Title, Text, URL, Keywords, Description; Searches HTML & XHTML pages, Atom, RSS & JSON feeds, and Plain text documents; Searches across Plugins: Bing, Google, Yahoo!; Results per Plugin: 1000 (max limit). The search terms entered were as follows: Perfectionism (anxiety OR depression OR stress OR trauma OR wellbeing OR “mental health” OR psycho*) (advice OR manag* OR treat* OR interven* OR prevent* OR strateg* OR tool* OR tip* OR help* OR assist* OR overcom*). Any websites that contained content on perfectionism were included. Excluded were websites that: were a peer-reviewed journal article, did not have enough relevant information, provided only a description/reiteration of published research, were therapy sites for resource dissemination. Personal blogs (n = 10) were included but their quality was not rated, as it was felt that these ratings could be interpreted as personal attacks on the individuals who wrote the blogs about their personal experience.

### Web site quality

Quality ratings were informed by the following elements: consistency with useful ideas, comprehensiveness, evidence-based information, empirically grounded strategies, and appropriate tone (not didactic, or talking down to people, providing an engaging read that provides the person with questions to consider application of the information to themselves). A 5-point Likert scale was created to capture these elements with the descriptors for each rating: Excellent (provides comprehensive, evidence-based information about perfectionism and empirically grounded intervention strategies, tone is appropriate), Good (reasonably comprehensive although not completely; most information provided is evidence-based, interventions are empirically grounded, tone is mostly appropriate), Moderate (somewhat comprehensive although large sections missing. Some evidence-based information but significant proportion is not empirically grounded, tone acceptable overall), Poor (inadequate information, information not evidence-based; strategies not empirically grounded, tone unacceptable), Potentially harmful. We randomly chose a subset of 20% of the websites to examine inter-rater reliability (54 of the 266 websites that were included in the final review) with respect to identified definitions and interventions.

### Web site theme extraction

Themes across the websites were extracted, summarising definitions of perfectionism and the strategies for managing perfectionism. An example from each descriptor/strategy from the website was recorded. These descriptors/strategies were then categorised by one author (MW) and examined by a second author (TDW); where differences in opinion existed, these were discussed and resolved. Inter-rater reliability for theme identification was calculated as follows: 1 = Themes and quotes highly consistent overall 2 = Themes consistent overall, some quote differences and/or greater variation in themes identified 3 = Themes and quotes identified predominantly different between raters.

### Youth advisory group survey

A youth advisory group comprising of 18 young people aged 18 to 24 (80% female) was formed from advertising through batyr, a for-purpose preventative mental health organisation in Australia created and driven by young people with lived experience of depression and anxiety. Young people were reimbursed with an AUD $80 Amazon voucher for 2 h of time. We provided the advisory group with the top five suggestions across the websites related to definitions, interventions, and the advantages and disadvantages of perfectionism, along with three questions addressing each: what was missing, what was most helpful, and what downsides were evident (see Table 3). Consultation comprised two stages (1) a Qualtrics survey and (2) an online group discussion using the Mentimeter platform.

**Table 3:** Survey questions for youth with lived experience of depression/anxiety in the Qualtrics survey

#	Top five suggestions across the websites	Question
	*Aspects of perfectionism* 1. Setting unrealistic high standards 2. Self-worth relies on reaching high standards 3. Unpleasant thoughts (Self-criticism, going over the same old thoughts again and again, worry that others don’t think you are any good, thinking you’re a failure if you make a mistake, all or nothing thinking (it is either “bad” or “good”), selectively attending to what you don’t do so well and ignoring your achievements 4. Feeling bad (Disappointment, shame, guilt, fear of failure, anxiety, depression, anger, stress, worry, frustration) 5. Counter-productive behaviours (Procrastination, checking your work over and over, invest lots of time in each task (can’t bear to let it go until it is “perfect”), avoid new challenges for fear of not doing well enough, over-committing, hyper-focus on order and details)	a. When you look at these descriptions, what do you think is missing? What would you add from your own experience? How do these affect each other? b. Which of these descriptions are most personally appealing to you? c. What, if any, are the downsides of these descriptions?
	*Strategies that can reduce perfectionism, depression and anxiety* 1. Identify thoughts and feelings 2. Develop alternative thoughts 3. Practising self-compassion not self-criticism 4. Procrastination 5. Judge your worth on things other than achievement	a. When you look at these strategies, what do you think is missing? What would you add from your own experience? b. Which strategies would be most helpful for reducing perfectionism? Why? Which of these strategies are most personally appealing to you? How do you think these strategies would help reduce anxiety and depression? c. What, if any, are the downsides of these strategies?
	*Advantages of Perfectionism* 1. Gives meaning, a sense of pride, achievement, challenge 2. Encourages self-improvement 3. Good at noticing mistakes 4. High levels of organisation 5. Good work ethic *Disadvantages of Perfectionism* a. Leads to stress and poorer mental health b. Workaholism c. Personal relationships and wellbeing suffer d. Negative impact on self-esteem e. Difficult to find genuine satisfaction	a. When you look at these advantages and disadvantages, what do you think is missing? What would you add from your own experience? b. Which advantage are most personally appealing to you? Why? Which disadvantage looks worst to you? Why? c. What, if any, are the downsides of knowing about these advantages and disadvantages?

## Results

### Website inclusion and quality

**Fig. 1:**
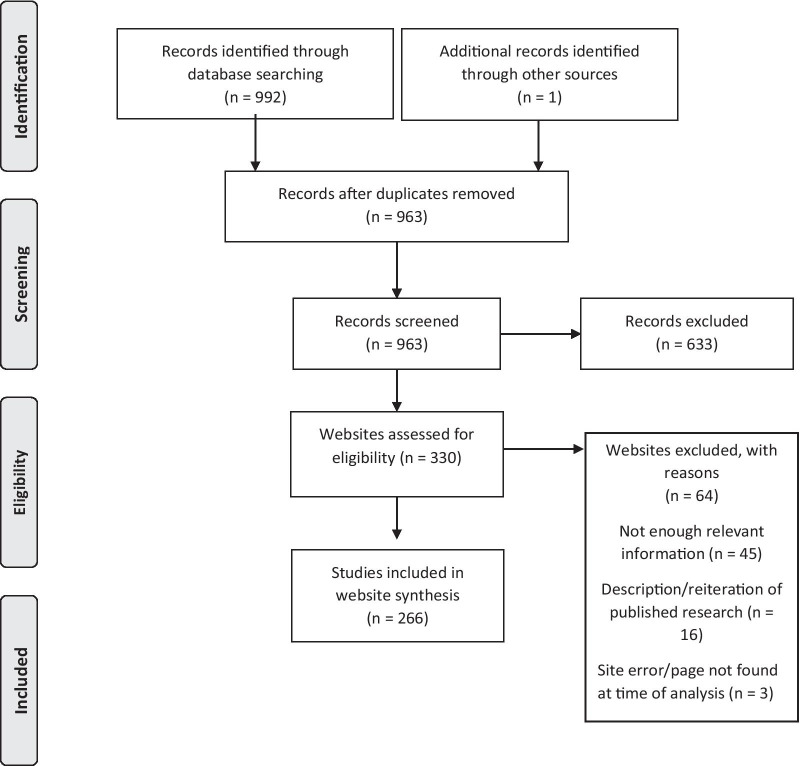
PRISMA flowchart for web synthesis

**Fig. 2:**
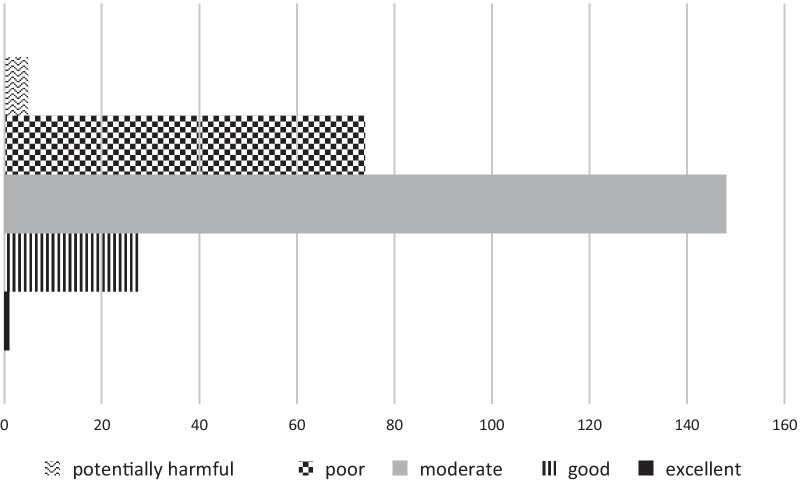
Ratings of the quality of 256 websites (excluding personal blogs) from 1 to 5 (mean = 3.21, SD = 0.67)

As shown in Fig. 1, the search yielded 992 websites, 266 of which were included in the qualitative synthesis. Generally, the informational websites were not interactive, but provided written opinions and some suggestions. The quality of the websites is depicted in Fig. 2, with only one website out of 256 (excluding personal blogs) meeting the criteria for excellent. The majority (56%) were judged as being of moderate quality. Five were considered potentially harmful for the following reasons: encouraging perfectionists to stay in detail-oriented role that “*play to their strengths*” and saying “*sometimes, a job or task needs to be perfect*”; advised to snap a rubber band every time you notice a critical thought; perfectionism can be a valuable asset and result in better outcomes; and justification of perfectionistic work habits and outputs. The kappa co-efficient for the quality rating was 0.461, which indicates a moderate level of agreement between the raters.

#### Definitions of perfectionism

Table 4 summarises the seven themes identified. Perfectionism was commonly recognised as consisting of behaviours, feelings and thoughts. Surprisingly, definitions of perfectionism were only offered in just over half the websites. Just under half the websites included information about the different domains of life impacted by perfectionism, the origins of perfectionism, and the idea that perfectionism could be both helpful and unhelpful.

**Table 4:** Descriptions of perfectionism identified across the 266 websites

Theme	N (%)	Examples
Definition of perfectionism	149 (56)	Rejects anything less than perfection; “suffocating pressure to meet every single expectation”; “devil on the shoulder whispering that you might fail if things aren’t flawless”; “tendency to set standards that are so high they either cannot be met or are only met with great difficulty”; “a combination of excessively high personal standards and overly critical self-evaluations”
Behaviours	234 (88)	“make lists of things they need to do ”, “ might become very controlling when it comes to relationships”, struggle to relax; procrastination, struggle to complete tasks, “avoiding taking the steps necessary to attain their desired outcomes”, working hard to “maintain an image of accomplishment and holding it all together”; deliberate self-harm
Feelings	252 (95)	body dissatisfaction, social phobia, excessive worry, chronic stress; fear of judgement, depression, anxiety, eating disorders, OCD, stress, escalating sense of pressure, anger; frustration, “continuous dissatisfaction with oneself”’; “I feel hollow, like I can never be happy”; “feel coming up short”; guilt, fear of failure, shame’ “deep seated unhappiness”
Thoughts	239 (90)	suicidal thoughts, fear over failure, scared of other's disapproval, self-critical; “This entails a lot of self-criticism, and that persecutory inner voice that constantly tells us how we could’ve done things better”; “impossibly harsh inner critic that gives you a bad review every time you do something”, “paralyzed by … not knowing where to start, fear of failing, …someone else's critiques”, “Not hearing what’s good about it anymore because you heard it too many times”, “Believing that it’s never going to be perfect”, “obsessing about the final outcome”
Origins	120 (45)	“childhood response to some form of trauma”; social media, “image driven, competitive world”; genes, learned behaviour; parental pressure, self-induced expectations, “competitive nature of most schools”
Dimensional nature	113 (43)	normal or adaptive perfectionism vs neurotic or maladaptive perfectionism … perfectionistic strivings and perfectionistic concerns; “perfection can be beneficial. It encourages us to always put in 110 percent. Keep in mind there’s also the dark side of perfectionism. Instead of wanting to do the best you can, you keep raising the bar higher and higher”
Domains of life affected	124 (47)	school, university, work; cleanliness, “house, career, body”, relationships, eating, exercise, creativity, performance; hobbies, appearance, parenting, entertaining; sports

#### Strategies to decrease perfectionism

Table 5 summarises the 15 strategies reaching our a priori threshold frequency at least 15% of the websites. The most common strategies across the websites were *identifying cognitions and developing alternatives*, *moving from self-criticism to self-compassion*, *normalising mistakes and seeing their benefit*, *adjusting goals, getting practical support*, and *tips about procrastination* and *time management*. The intervention strategies map closely to the CBT developed for perfectionism and shown to be effective in youth. New information, however, was introduced in terms of a greater emphasis on getting practical support.

**Table 5:** Descriptions of effective interventions for perfectionism identified across the 266 websites

Theme	N (%)	Examples
Pros and cons of perfectionism and change (why is perfectionism maintained)	78 (29)	Identify the hidden payoff in perfectionism, get radically honest about the costs; Evaluate the costs and benefits of spending a large amount of time making sure things are just so
Differentiating perfectionism from striving for high standards	54 (20)	Striving to do your best and always push the envelope is great. It’s the force that motivates you to keep going and improve your craft. But when it turns into perfectionism, it becomes a blocker
Psychoeducation about perfectionism	45 (17)	
Expanding self-evaluation	66 (25)	Value Yourself: You’re more than a percentage, you’re far from a pile of imperfections and there is no such thing as ‘perfect’. We’re all different for a reason, each with our own unique gifts to offer. Show the world your talents, explore your passions and let the inner you shine
Moving from self-criticism to self-compassion	97 (37)	Forgive yourself for your shortcomings. Nobody is perfect, and everybody has strengths and weaknesses; If you’d be happy with your performance if it had come from someone else, then give yourself the recognition you deserve
Self-monitoring	56 (21)	Changing a habit takes practice. The first step is mindfulness — that is, becoming aware of a painful habit, such as always comparing yourself to others; Awareness is always the first step … get to know the negative inner voice: What does it say, when does it show up? Does it remind us of someone familiar? Is there any bodily sensation that goes with it?
Thinking errors	63 (24)	All-or-Nothing/Black and white thinking
Identifying hot cognitions and identifying alternatives	98 (37)	Identify and rationally challenge our [perfectionist] thoughts in order to see why we’re getting so bothered: 'Is my way the only way to view this situation? Would another person necessarily see this situation the same way as I do?' … “notice your perfectionist thoughts, generate alternative thoughts, choose a more realistic way to look at the situation
Behavioural experiments	65 (24)	Hypothesis testing of the accuracy of your perfectionistic thoughts and predictions. Try pressing “send” on that email without proofreading it. Try showing up five minutes late for that meeting. Try buying pants online without reviewing every option available … “Regardless of the outcome, you will obtain valuable information. If there is no consequence, you will learn that your beliefs about the importance of including all of the details are not true” … “next time you are asked a question and don’t know the answer, just say, “I don’t know.” Then keep track of how many friends you lose. See how much less loved you are. Note particularly how much less respect you get
Procrastination and time management	81 (31)	Create “good enough” deadlines for yourself … don’t allow yourself to check something “one last time.” Set deadlines that let you move on so you don’t get stuck trying to perfect one project at the expense of another … determine how long a project will take to complete. Aim for efficiency and to finish in 5% less time than you initially allocated for the task, and don’t let yourself go over by more than 10% … limit the [distractions] you have control over (like taking a quick break to scroll through your Instagram feed)
Get practical support	86 (32)	Therapist, connect with others, create “safe environment to explore new things and move forward”, get others involved when too stuck on the project to see clearly
Adjusting goals and expectations	87 (33)	Consider loosening those standards” … Re-set your standards in steps, eg. Gradually take less and less time to prepare for presentation, or being ok with fewer and fewer people praising your performance; Encouraging flexibility: “Teach your students that if plan A doesn’t work there are 25 more letters
Mistakes: normalising and seeing the benefit	92 (35)	knowing that we make mistakes as humans, but we live and learn; create a classroom culture where it is ok to fail. Our students need to be reassured that failure is the key to success; trial and error are essential for learning and achievement
Prioritising process over outcome	47 (18)	Celebrate the journey, not the outcome. Even if you don’t manage to hit a target or achieve something you wanted to, focus on the effort you put into it and what you learnt along the way; Evaluate your success not only in terms of what you accomplished but also in terms of how much you enjoyed the task. Recognize that there can be value in the process of pursuing a goal
Mindfulness and gratitude	43 (16)	Practice mindfulness: Increase your self-awareness through mindfulness exercises. Mindfulness can allow you to come to terms with your thoughts about perfectionism, making you more aware of your perfectionistic tendencies and allowing you to face these thoughts without reacting to them. Through the practice of mindfulness, you can learn to let go and release the stress associated with perfectionism

#### Advantages and disadvantages of perfectionism

The top five advantages and disadvantages of perfectionism are shown in Table 3, with achievement and organisation being highly endorsed as advantages. Damage to mental health and wellbeing and social relationships were recognised as disadvantages, as was the inability to be satisfied or feel good about achievement.

#### Inter-rater agreement on descriptions and strategies

The informational themes were consistent for 100% of the websites (54% highly consistent and 41% with some variation). The agreement for the strategy themes was lower, 71% with consistent themes (36% highly consistent and 35% with some variation), and 29% with disparate themes.

### Youth advisory group consultation

We obtained 12 complete Qualtrics survey responses and 6 survey responses that were partially complete (3 completed only first 2 questions about perfectionism, and another 3 stopped responding after 'Area 1: how would you describe perfectionism'). Subsequently, one online Mentimeter group was held consisting of four females, two males, and two participants with no nominated gender, using the same survey questions. Very little new information was generated using this format, and hence the feedback received between both the survey and group discussion were integrated.

#### Definitions of perfectionism

**Fig. 3:**
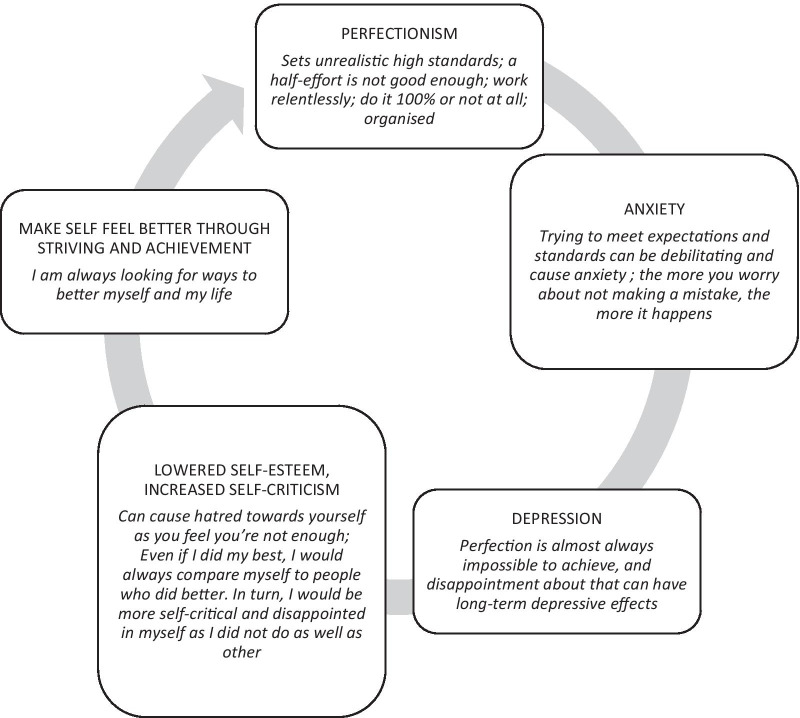
Conceptual model of perfectionism informed by young people with lived experience of anxiety and depression

The majority of responses to the question about what was missing involved adding a greater description of unpleasant emotion associated with perfectionism e.g., “*go more in depth with the “feeling bad” section because from personal experience I wouldn't say I “feel bad” more just feeling so stressed that the situation or the task makes me so uncomfortable, causing me to become unfocused”, “More mention of anxious feelings”*. Young people described the vicious cycle whereby perfectionism led to feelings of depression and anxiety, which decreased self-esteem and increased self-criticism, leading to a drive to feel better through achieving perfectionistic standards. The most helpful ingredient was identified as setting more realistic standards, as this offered most hope for change e.g., “*can be used to help to reduce perfectionism because you can actively work on setting attainable and realistic goals”, “seems the most easily “changeable” to me, whereas it takes a lot more work to change one's mindset*”. Eight young people said that they found the link between goals and self-worth to be the most unpleasant part of perfectionism e.g., “*because sometimes when I make goals for myself, I can become very depressed if I am unable to achieve them rather than telling myself I can do better next time”, “low self-worth is a factor in depression, and having a factor as important as self-worth riding on the near impossible feat of achieving perfection creates anxiety”, “linked to depression*”. This feedback was used to develop a vicious cycle of perfectionism, summarised in Fig. 3. The cycle recognised the primacy of anxiety about achieving when perfectionism was present, which can then translate to depression when people feel not good enough, which then results in self-criticism, lowered self-esteem, and an intensification of efforts to achieve in order to feel better about oneself.

#### Strategies to decrease perfectionism

Responses are summarised in Table 6, where the strategy most considered missing (27%) was seeking social support. The same proportion of people identified a related downside of the outlined strategies, namely that they were difficult to complete. The most appealing strategies included alternative thoughts, practicing self-compassion, judging self-worth on things other than achievement, and dealing with procrastination. It should be noted, however, that this latter strategy was particularly identified by one person as being unhelpfully simplistic.

**Table 6:** Youth responses to the questions about helpful strategies for reducing perfectionism (N = 15)

Questions and responses	N
When you look at the strategies in the pamphlet, what do you think is missing?	
Seeking social support is important e.g., “*More strategies that involve others because sometimes I find it hard to use the strategies in the pamphlet when I am in a state of mind causing me to believe that I am not doing good enough*”	4
Which strategies are most helpful/appealing?	
Alternative thoughts e.g., “*I like to look at situations in an objective way when I find myself getting so emotional that it causes me to have anxious or depressive thoughts. This way I am able to think more calmly and rationally about a situation and “solve it” as well as possible*”	5
Practicing self-compassion, e.g., “*something that my psychologists and support network have encouraged me to do … directly related to reducing anxiety and depression; it is an important part of self-care”*	5
Judging self-worth on things other than achievement, e.g., “*Make a list of the qualities you like about yourself, the wonderful relationships you have, and positive experiences you have had. Very easy to lose sight of the things you cherish most in life when you have access to them*”	3
Dealing with procrastination, e.g., “*I tend to not want to start things because of fear it won’t meet the standards I have set I think breaking things up and moving on from things when they are not how I want would help me be less stressed and anxious*”	3
What are the downsides of the strategies?	
They are unrealistic/hard to do e.g., “*Practicing self-compassion is a really difficult thing to do, and something that many people will feel silly doing or not be able to do, and as a result will not practice it”; “The dealing with procrastination one made me angry just reading it. It seems extremely unrealistic. I would never do that”; “These strategies are not an “easy fix*”; *they need to be practiced and stuck with for a period of time in order to have an effect*”	4
No downsides	3

#### Advantages and disadvantages of perfectionism

Responses indicated that perfectionism could work very well or very badly for the same person e.g., *I sometimes find that my perfectionism can cause me to either hyper focus on an activity or shut down to the point that I cannot do the activity at all.* The advantages that were emphasised included *self-improvement, organisation, good work ethic, critical thinking, the endorphins associated with the feeling of succeeding and completing assignments, investment in meaningful domains of life, and productivity*.

## Conclusions

This realist synthesis identified most of the websites were of moderate quality. Only around half of the websites contained empirically supported information about definitions and, to a much lesser extent information about strategies and interventions, and advantages/disadvantages. While the general tone was acceptable, the websites were not highly engaging or likely to promote change, including help-seeking. Young people examining the messages on the websites identified that further emphasis on the relationship between depression, anxiety and perfectionism is required, and that strategies and interventions were difficult, requiring recognition that greater levels of support are needed to put the strategies into action.

### Implications for theory

There are several possible routes by which perfectionism operates to contribute to the development and maintenance of anxiety and depression in youth; the specific mechanism of action is likely to vary for different people according to their individual situations. The conceptual model developed across the information and websites and from the feedback from youth suggested that perfectionism results in increased levels of anxiety about performance, which then leads to depression about the difficulties of reaching desired standards, which can lead to withdrawal and social isolation, associated with lower self-esteem, and increased self-criticism. Consistent with the clinical perfectionism model [3], this leads to an increased focus on striving/achievement in order to repair damaged identity through external validation of achievement.

Maintenance of the vicious cycle was attributed to three factors. Almost half the websites discussed the importance of reinforcing contexts, including parents, teachers or peers who place pressure to attain high standards in different domains of life (e.g., study, appearance, sport), social media, and educational environments. Some websites and most youth identified perceived benefits of perfectionism, for example including feeling good about success, working hard and organisation. These perceived benefits pose a challenge in engaging people in an intervention. A third major contributor to maintenance was that change is viewed as being very hard and that it was unrealistic to expect change without support, particularly in the complex differentiation of moving towards healthy high standards versus perfectionism.

### Practical implications

First, informational websites should consider including content on the most endorsed evidence-based strategies. These include introduction of domains of life that are not reliant on achievement but that contribute to our identity (i.e., make us feel good when going well and make us feel bad when not going well) to provide competition to self-evaluation being overly influenced by achievement; modifying rigid all-or-nothing goals to more realistic and flexible guidelines, with mistakes celebrated as the pathway to better performance; psychoeducation about the utility of self-compassion in terms of productivity [20]; overt identification of the perceived positive aspects of perfectionism and challenging whether the outcomes can be achieved in different ways that do not result in negative impacts. Second, internet treatments should consider provision of some type of support such as guided self-help, which is generally shown to produce better results than unguided internet interventions [21]. Third, the findings also speak to the importance of policies to govern the internet. A growing field in its own right [22], the results highlight the need for mental health websites such as those addressing perfectionism to be governed appropriately.

### Limitations

The use of realist synthesis has been criticised [17] both in terms of a lack of methodological clarity and insufficient distancing from methodology influenced by using inferences of causality from standard experimental studies. We also recognise that the youth commenting on the websites may not have necessarily engaged in websites related to perfectionism, and feedback from a group accessing these websites for their own needs may have enriched the informativeness of our outcomes. The invitation to participate was sent to batyr who then forwarded it on to their members, so we do not know how many people received the invitation, and are unable to calculate response rates or whether these participants are representative of youth who have experienced perfectionism which results in anxiety and depression. We also note that the use of the Mentimeter platform (i.e., not face to face) and using the same questions as the survey was not a useful addition to our information gathering. This research was conducted during the COVID-19 pandemic making face-to-face groups difficult, but future research would benefit from using face-to-face focus groups in terms of generating new insights.

## Conclusions

We conclude that people should be wary of relying on current internet information about perfectionism. Although the content was moderately accurate, youth with lived experience were clear that this was not a replacement for psychosocial support to work through interventions. We suggest that websites might improve their information in the following ways. First, the quality of internet websites addressing perfectionism needs to improve, as they currently contain very little evidence-based advice. The role of perfectionism in promoting anxiety and depression needs to be further emphasised, allied with clear links to current strategies and interventions shown to decreases perfectionism and improve mental health and wellbeing in youth. Second, more evidence of co-design in the content of websites needs to occur, such that the information and strategies provided align with the experience of young people, thus aiding in better engagement and more effective dissemination of strategies. Related to this, multi-language formats and formats that address cultural and gender diversity are required. Third, prevention and treatment programmes need to be more active in focusing on helping young people develop support networks that can help them find their way between unhealthy perfectionism and healthy striving for excellence and valued goals. Future research should examine whether making such changes to online information and interventions results in greater levels of engagement and help-seeking and improvements in anxiety and depression in young people.

## Data Availability

The data that support the findings of this study are available from the corresponding author upon reasonable request.

## Abbreviation

CBT-P: Cognitive behaviour therapy for perfectionism
